# Evaluation of renal ischemia–reperfusion injury using CEUS in mice

**DOI:** 10.1186/s41747-023-00392-3

**Published:** 2023-12-19

**Authors:** Zixin Xu, Xinbao Zhao, Ming Huang, Qi Liu, Libo Liu, Junjiong Zheng, Chao Fang, Wen Dong

**Affiliations:** 1 Department of Urology, Guangdong Provincial Key Laboratory of Malignant Tumor Epigenetics and Gene Regulation, Sun Yat-Sen Memorial Hospital, Sun Yat-Sen University, Guangzhou, China; 2 Department of Ultrasound, Guangdong Provincial Key Laboratory of Malignant Tumor Epigenetics and Gene Regulation, Guangdong-Hong Kong Joint Laboratory for RNA Medicine, Sun Yat-Sen Memorial Hospital, Sun Yat-Sen University, Guangzhou, China

**Keywords:** Contrast media, Ischemia, Kidney, Reperfusion injury, Ultrasonography

## Abstract

**Background:**

Renal ischemia–reperfusion injury (IRI) frequently occurs clinically. We investigated the value of contrast-enhanced ultrasonography (CEUS) in the evaluation of renal IRI levels in mice.

**Methods:**

Thirty-six healthy adult male C57BL/6 mice (20–22 g) were randomly divided into the sham, 10 min, 20 min, 30 min, 40 min, and 50 min groups based on the time of renal warm ischemia by blocking the left renal pedicle, approved by the Institutional Animal Ethics Committee. Time-intensity curve (TIC)-derived parameters such as peak enhancement (PE) and wash-in perfusion index (WiPI) were produced using CEUS at 1 h and 24 h after IRI. The severity of kidney injury was detected by the renal tubular necrosis rate which was analyzed by hematoxylin and eosin staining at 24 h after IRI. The Spearman correlation coefficient was used to describe the correlations between PE and WiPI values and the renal tubular necrosis rate.

**Results:**

The PE and WiPI values decreased after IRI in the groups with a warm ischemia time ≥ 20 min. The renal tubular necrosis rate was significantly correlated with the PE value at 1 h (ρ = -0.802) and 24 h (ρ = -0.861) after IRI and the WiPI value at 1 h (ρ = -0.814) and 24 h (ρ = -0.853) after IRI (all *p* < 0.001).

**Conclusion:**

TIC-derived parameters, including PE and WiPI values, can be used to evaluate the severity of renal IRI in mice. CEUS is a safe and effective technology for the detection of renal IRI.

**Relevance statement:**

CEUS can evaluate the severity of renal ischemia–reperfusion injury by peak enhancement and wash-in perfusion index values selected from various time-intensity curve-derived parameters.

**Key points:**

• Contrast-enhanced ultrasonography can evaluate the level of renal ischemia–reperfusion injury.

• Peak enhancement and wash-in perfusion index are correlated with the renal tubular necrosis rate.

• CEUS can detect changes in unilateral renal function without radiation.

**Graphical Abstract:**

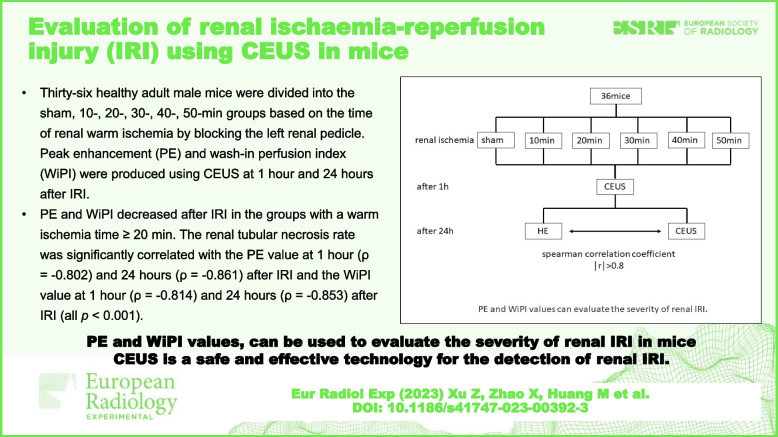

**Supplementary Information:**

The online version contains supplementary material available at 10.1186/s41747-023-00392-3.

## Background

Partial nephrectomy is a standard treatment option for the management of cT1 and cT2 renal tumors when technically feasible. The renal artery is clamped during surgery, which can lead to ischemia–reperfusion injury (IRI). It is traditionally thought that the maximum time of renal warm ischemia is 30 min [1]. However, some other studies reported that the safe time for warm ischemia was less than 20 min [2–4]. The maximum time of warm ischemia without potential influence on renal function is still controversial.

Patients who experience IRI may have acute kidney injury (AKI) postoperatively. Acute tubular injury and acute tubular necrosis are the major causes of AKI [5, 6]. Current methods of assessing renal function mainly rely on serum creatinine, blood urea nitrogen, and radionuclide dynamic renal imaging. Serum creatinine and other indicators cannot be used to evaluate unilateral renal function. Radionuclide dynamic renal imaging can be used to detect split renal function and evaluate the severity of renal IRI [7]; however, it involves radiation. Currently, we lack effective methods to evaluate the severity of renal IRI. We hope to find a new technique to evaluate unilateral renal function.

Contrast-enhanced ultrasonography (CEUS) is an ultrasound method that allows real-time evaluation of various structures at different vascular stages [8]. Highly echogenic microbubbles are used as contrast media to increase the sensitivity and specificity of blood flow measurements [9]. The ultrasonic echo signal at the same location is enhanced, according to which a time-intensity curve (TIC) is drawn. TIC-derived parameters such as peak enhancement (PE), area under the curve (wash-in) (WiAUC), rise time (RT), time to peak (TTP), wash-in rate (WiR), and wash-in perfusion index (WiPI), *i.e.*, the WiAUC/RT ratio, can be utilized to reflect the blood flow status [10]. CEUS is used to diagnose renal infarction, inflammatory renal lesions, and parenchymal causes of early graft dysfunction [11]. CEUS allows dynamic and noninvasive detection of renal perfusion impairment after ischemic AKI [12]. However, it is still unclear which TIC-derived parameters can accurately reflect the severity of renal IRI.

In this study, we used CEUS to assess the blood flow of unilateral kidneys after renal IRI in mice. The aim of this experimental study was to assess the severity of IRI by TIC-derived parameters and explore the safe time for renal warm ischemia.

## Methods

### Animals and groups

The animal experiments were approved by the Institutional Animal Ethics Committee (AP20230045). Thirty-six healthy adult male C57BL/6 mice (20–22 g) were obtained from Guangzhou Ruige Biological Technology Co., Ltd. The animals were accommodated for a week before the surgical procedure with water and food. The mice were randomly allocated to one of the following groups: the sham group (*n* = 6); the 10-min group (*n* = 6); the 20-min group (*n* = 6); the 30-min group (*n* = 6); the 40-min group (*n* = 6); and the 50-min group (*n* = 6). The clamp was released after the left renal pedicle of mice in the 10-min group was clamped for 10 min. The other experimental groups underwent analogical treatment.

### Mouse renal IRI model

All mice were anesthetized by inhalation of 1.2% isoflurane and placed on a heating pad. Surgical preparation for the left renal area included hair removal and betadine disinfection. The body surface projection of the mice’s left kidneys was determined by two-dimensional ultrasound. The mice were placed in the operation position. A longitudinal incision on the left side of the back was made and back skin and tissue were separated layer by layer. The left renal pedicle was clamped with a nontraumatic vascular clamp. After the corresponding time, the vascular clip was released and the tissue and skin were sutured layer by layer. The left renal pedicle of the sham group was isolated except for clamping. Animals had access to water and food after surgery.

### CEUS protocol

Meng’s method was followed for CEUS [13]. The mice were anesthetized and secured before surgery, 1 h after surgery, and 24 h after surgery. The mouse kidneys were examined with an animal ultrasound machine for at least 3 min immediately after the injection of the contrast agent to obtain a complete TIC curve. According to the manufacturer’s instructions (Visualsonics, Tokyo, Japan), high-resolution ultrasound imaging of the kidneys was performed using the Vevo® 2100 system (Fujifilm Visualsonics, Tokyo, Japan) with an MS250 transducer. Blood flow and kidney perfusion measurements were performed with CEUS according to the manufacturer’s instructions (Vevo® 2100 system). The mice were injected with a 100 μL bolus of Vevo Micromarker suspension (Visualsonic) via the tail vein using a MicromarkerTM catheter. Using the VevoCQTM contrast quantification software, ultrasonic images were processed and analyzed according to the manufacturer’s instructions. The TIC was drawn and derived parameters such as PE, WiAUC, RT, TTP, WiR, and WiPI were collected. The TICs of the regions of interest (ROIs) were then processed and analyzed.

### Renal function assessment

After CEUS, 2.0 mL of blood was extracted by enucleation of the eyeball after anesthesia and centrifuged at 3,000 rpm at 25 ℃ for 10 min. The supernatant was collected and submitted to the Clinical Laboratory of Sun Yat-sen Memorial Hospital of Sun Yat-sen University for the measurement of serum creatinine, blood urea nitrogen, and cystatin C.

### Histologic evaluation

After blood extraction, the mice were euthanized. Part of the tissue of the left kidney was fixed in 4% neutral buffered paraformaldehyde solution, paraffin-embedded, sectioned, and stained with hematoxylin and eosin, and periodic acid-Schiff. These staining results were evaluated by the Department of Pathology of Sun Yat-sen Memorial Hospital of Sun Yat-sen University.

### Statistical analysis

IBM SPSS Statistics 26 was used for data analysis. Numerical data are expressed as the mean ± SD. The comparison between groups was performed by ANOVA, followed by the LSD test when *p* > 0.05 in the homogeneity test of variance or Dunnett's T3 test when *p* < 0.05 in the homogeneity test of variance. The Spearman correlation coefficient was used to describe the correlation among parameters. A *p* value lower than 0.05 was considered statistically significant.

## Results

### Tubular necrosis after renal IRI

**Fig. 1:**
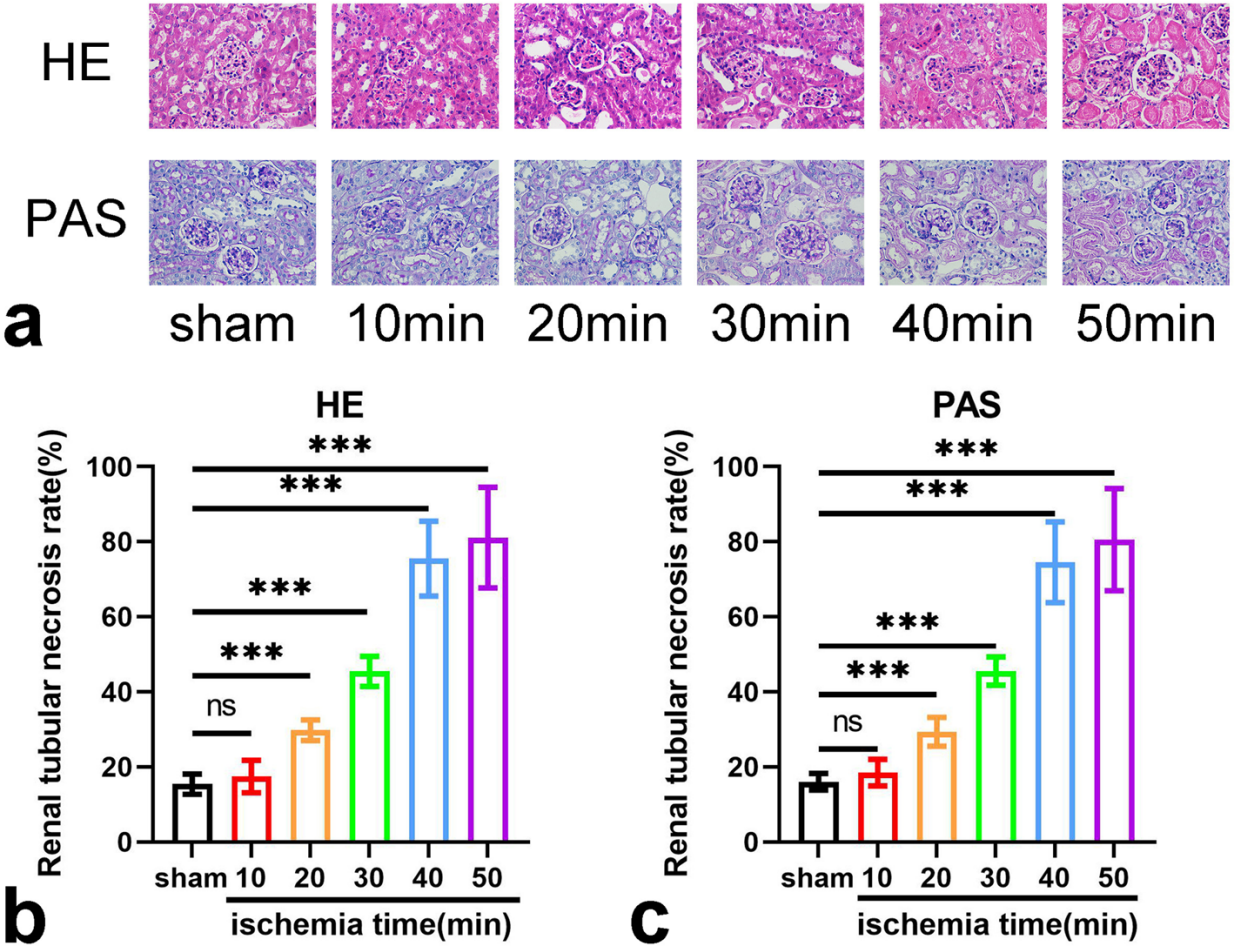
The histopathological examination of kidneys after ischemia–reperfusion injury. **a** The histopathological examination of kidneys of each group by HE staining at 24 h after IRI (magnification × 400) and PAS staining at 24 h after IRI (magnification × 400). **b** The renal tubular necrosis rate by HE staining (%). **c** The renal tubular necrosis rate by PAS staining (%). The renal tubular necrosis rate (%) = necrotic renal tubules / total renal tubules × 100%. *Ns* No significance, *p* > 0.05; ***, *p* < 0.001. *HE* Hematoxylin and eosin, *PAS* Periodic acid-Schiff

After clamping the left renal pedicle of mice for 10~50 min, we found that a short clamping time (≤ 10 min) did not cause detectable tubular necrosis. A longer time (≥ 20 min) could cause acute tubular necrosis. The severity of renal IRI increased gradually with prolonged warm ischemia time (Fig. 1a–c).

### Effect of blocking blood supply on serum creatinine, blood urea nitrogen, and cystatin C

**Fig. 2:**
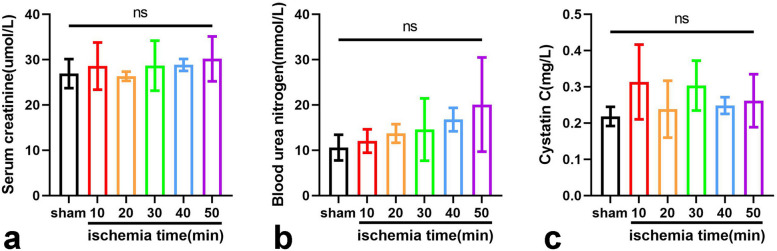
Serum creatinine (**a**), blood urea nitrogen (**b**), and cystatin C (**c**) levels in the six groups of mice. *Ns* No significance (*p* > 0.05)

The serum creatinine levels in the 10-min group, the 20-min group, the 30-min group, the 40-min group, and the 50-min group were 28.58 ± 5.20 µmol/L (mean ± standard deviation), 26.33 ± 1.03 µmol/L, 28.67 ± 5.50 µmol/L, 28.83 ± 1.33 µmol/L, and 30.17 ± 4.96 µmol/L, respectively, which were not statistically significant compared to the sham group (Fig. 2a). The blood urea nitrogen levels in the above five groups were 12.03 ± 2.58 mmol/L, 13.70 ± 2.06 mmol/L, 14.57 ± 6.90 mmol/L, 16.77 ± 2.57 mmol/L, and 20.08 ± 10.41 mmol/L, respectively (Fig. 2b). The cystatin C levels in the above five groups were 0.31 ± 0.10 mg/L, 0.24 ± 0.08 mg/L, 0.30 ± 0.07 mg/L, 0.25 ± 0.02 mg/L, and 0.26 ± 0.07 mg/L, respectively (Fig. 2c). Neither blood urea nitrogen nor cystatin C was statistically significant compared to the sham group. These results indicated that there were no significant changes in serum creatinine (*p* = 0.600), blood urea nitrogen (*p* = 0.068), and cystatin C (*p* = 0.140) after unilaterally blocking the unilateral renal blood supply.

### Decrease in CEUS PE and WiPI after IRI

**Fig. 3:**
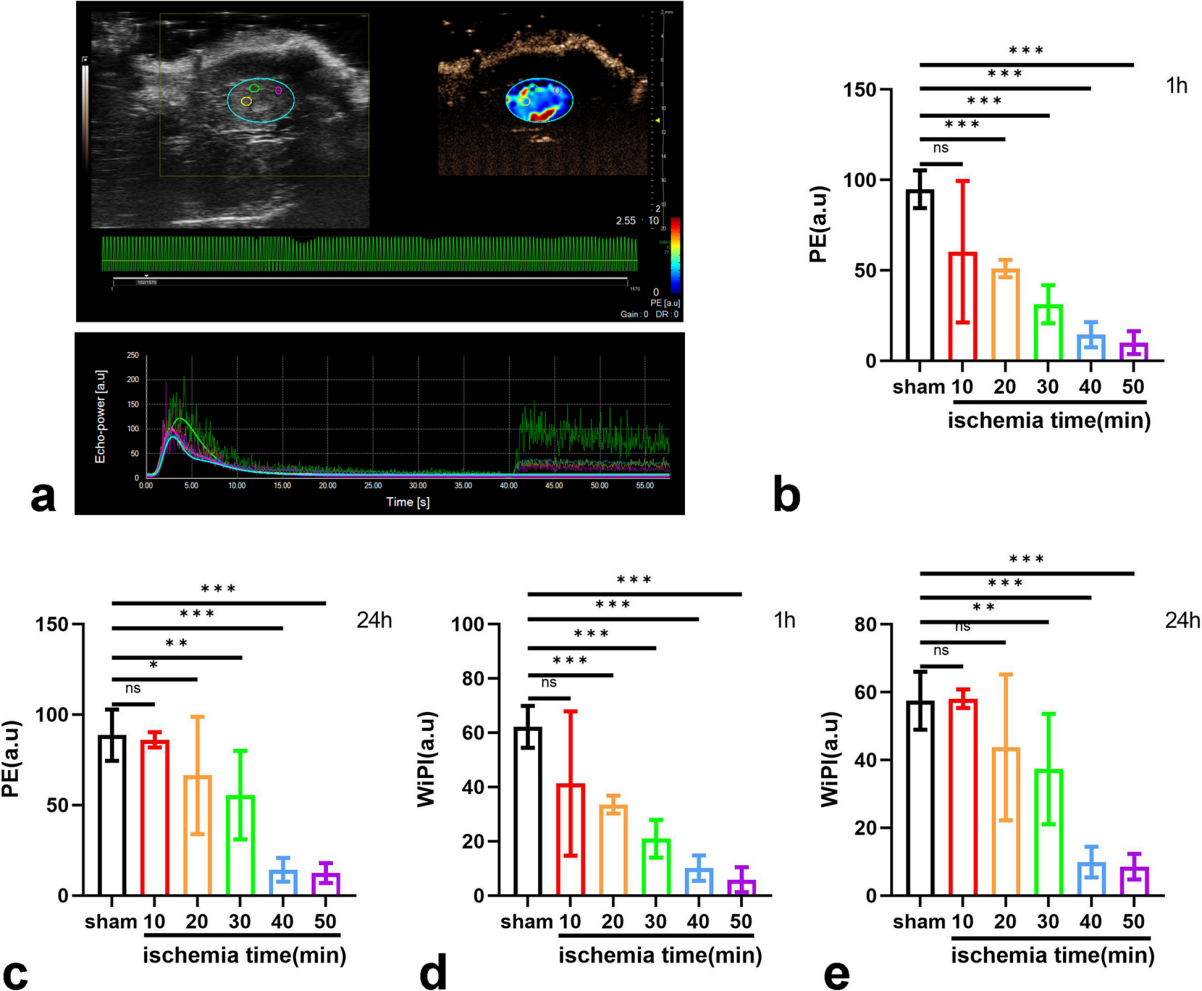
The CEUS image and the TIC image after IRI. **a** The CEUS image and the TIC image of the sham group 1 h after IRI. The green, yellow, and purple annular parts were three randomly selected sites in the renal cortex in the image, labeled as ROI 1, ROI 2, and ROI 3. The blue annular part was the location of the kidney in the image, labeled as ROI 4. PE values 1 h (**b**) and 24 h (**c**) after IRI. WiPI values 1 h (**d**) and 24 h (**e**) after IRI. The value for each mouse was calculated as the mean of the values of ROI 1, ROI 2, and ROI 3. *Ns* No significance, *p* > 0.05; *, *p* < 0.05; **, *p* < 0.01; ***, *p* < 0.001. *a.u.* Arbitrary units, *IRI* Ischemia‒reperfusion injury, *PE* Peak enhancement, *ROI* Region of interest, *WiPI* Wash-in perfusion index

After the injection of the contrast agent via the tail vein in the mice, the renal arteries, cortex, and pyramid were clearly displayed, which was shown as a “fireball-like” signal enhancement. Signal intensity peaked fleetly in the cortex and then gradually decreased to baseline. The kidney images and TIC images of CEUS are displayed (Fig. 3a, Figure S1a). The PE values declined 1 h after IRI compared to the sham-operation group (*p* < 0.001). The PE values were still smaller than those of the sham-operation group 24 h after IRI (*p* < 0.001) (Fig. 3b, c). The WiAUC values of the 40-min group (*p* = 0.006) and the 50-min group (*p* = 0.001) were smaller than that of the sham-operation group 24 h after IRI (Figure S1b, c). There was no significant difference in the RT values and TTP values among all groups (Figure S1d–g). A significant reduction was observed in the WiR in the 30-min group (*p* = 0.049) and the 50-min Group (*p* = 0.030) 1 h after IRI; however, it was not observed 24 h after IRI (Figure S1h, i). The WiPI values declined when the warm ischemia time was ≥ 20 min 1 h after IRI (*p* < 0.001) and when the warm ischemia time was ≥ 30 min 24 h after IRI (*p* < 0.001) (Fig. 3d, e). These results suggested that PE values and WiPI values decreased after IRI in the groups with a warm ischemia time ≥ 20 min. The WiAUC values and WiR values could be observed to decline when the WI time was long; however, the results were not highly consistent in all groups when the warm ischemia was more than 30 min. There were no significant differences in the RT values and TTP values between the ischemia groups and the sham-operation group.

### Correlation between TIC-derived parameters and tubular necrosis

**Fig. 4:**
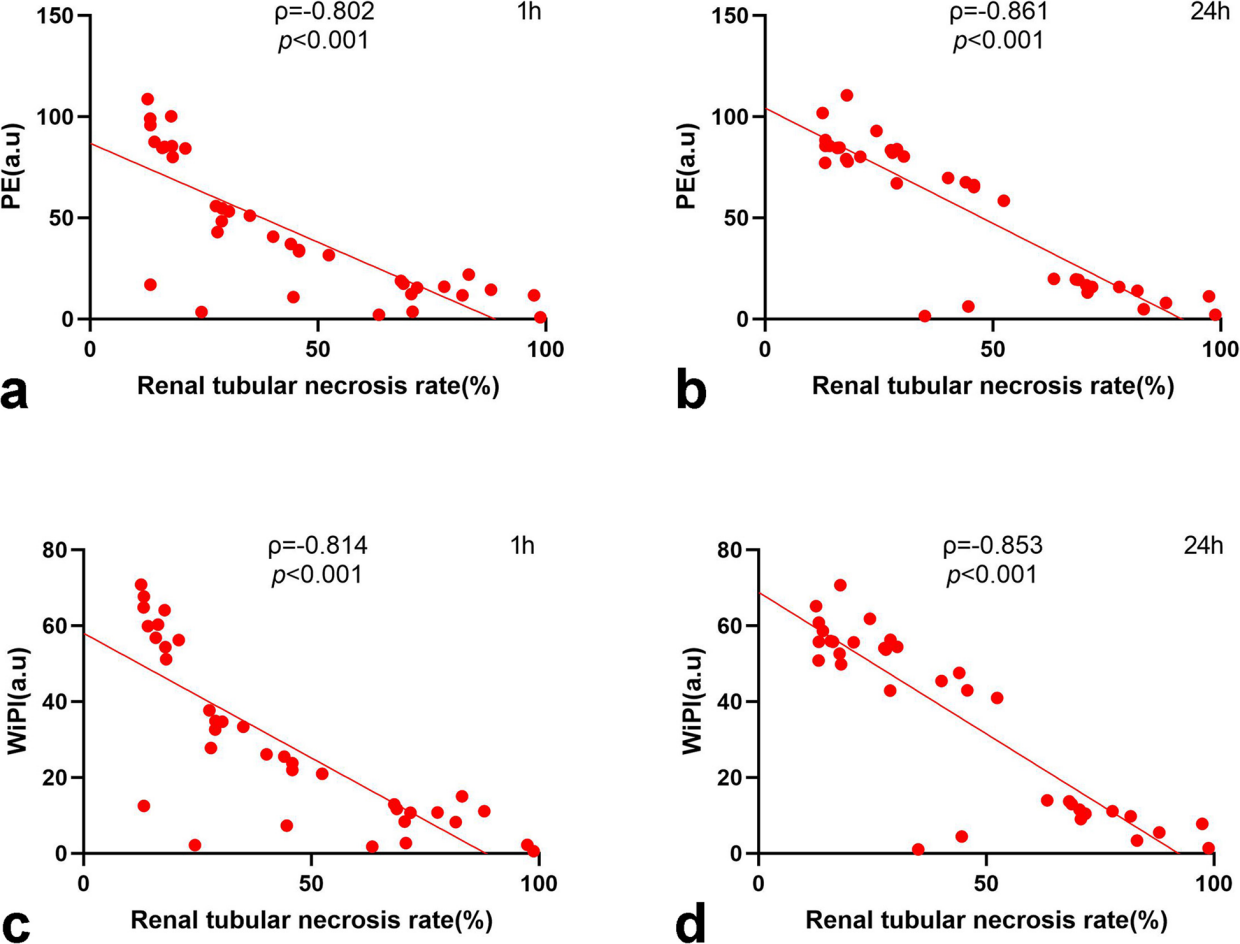
The scatter plots of the TIC-derived parameters and the renal tubular necrosis rate by HE staining (%) at 24 h after IRI. **a** Scatter plot of PE (a.u) at 1 h after IRI and the renal tubular necrosis rate (%). **b** Scatter plot of PE (a.u.) at 24 h after IRI and the renal tubular necrosis rate (%). **c** Scatter plot of WiPI (a.u.) at 1 h after IRI and the renal tubular necrosis rate (%). **d** Scatter plot of WiPI (a.u.) at 24 h after IRI and renal tubular necrosis rate (%). *a.u.* Arbitrary units, *IRI* Ischemia–reperfusion injury, *PE* Peak enhancement, *WiPI* Wash-in perfusion index

To explore whether CEUS could be used to evaluate the severity of renal injury after IRI, the Spearman correlation coefficient was used to describe the relationship between all parameters. The renal tubular necrosis rate was significantly associated with the PE, WiAUC, RT, TTP, WiR, and WiPI values at 1 and 24 h post-IRI (Fig. 4a–d, Figure S2a–h and Table 1). There were strong correlations between the PE and WiPI values and the renal tubular necrosis rate (|ρ|> 0.8) (Fig. 4a–d). These results showed that the changes in the PE and WiPI values could reflect the severity of kidney injury. The two values at 1 h and 24 h post-IRI indicated the consistency of the same results even at different time points to reflect AKI.

**Table 1:** Correlation between TIC-derived values and renal tubular necrosis rate

	Necrosis rate 1 h after IRI	Necrosis rate 24 h after IRI
Peak enhancement		
ρ	-0.802^***^	-0.861^***^
*p*	< 0.001	< 0.001
Wash-in area under the curve		
ρ	-0.459^**^	-0.597^***^
*p*	0.005	< 0.001
Rise time		
ρ	0.467^**^	0.455^**^
*p*	0.004	0.005
Time to peak		
ρ	0.409^*^	0.361^*^
*p*	0.013	0.030
Wash-in rate		
ρ	-0.694^***^	-0.664^***^
*p*	< 0.001	< 0.001
Wash-in perfusion index		
ρ	-0.814^***^	-0.853^***^
*p*	< 0.001	< 0.001

*IRI* Ischemia–reperfusion injury

^*^p < 0.05; ^**^p < 0.01; ^***^p < 0.001

## Discussion

This study demonstrated that TIC-derived parameters from CEUS could reflect the severity of renal IRI. We found that in mice the PE and WiPI values significantly decreased when the warm ischemia time reached 20 min, which was consistent with findings of acute tubular necrosis observed pathologically 24 h after renal IRI. The two values also had consistency at different time points to reflect AKI.

One study showed that CEUS enabled the evaluation of renal perfusion impairment associated with chronic kidney disease after ischemia AKI, and indicated that CEUS may serve as a noninvasive technique for assessing AKI to chronic kidney disease progression [12]. However, that study did not explore which parameters of CEUS could be used to reflect the severity of AKI. In our study, we showed that PE and WiPI values, which were selected from TIC-derived parameters, could accurately reflect the severity of renal IRI.PE and WiPI values are analyzed by CEUS images. As cortical blood flow takes up most of the whole renal blood flow, the renal cortex can be used as an ROI to analyze the renal blood perfusion [14]. Three ROIs were chosen to analyze and take the average values of TIC-derived parameters to reduce the error. PE was calculated at the time of maximum enhancement in the ROI. It was measured during the complete filling of the blood vessel with microbubbles [15] and related to fractional blood volume [16]. WiPI is the ratio of WiAUC to RT (WiAUC/RT). It provides a single number representing tissue perfusion [17]. We observed that PE and WiPI values decreased as renal blood perfusion was reduced after IRI.

CEUS is superior for detecting split renal injury post IRI to other tests including haematological tests and radionuclide dynamic renal imaging. Hematological tests, such as serum creatinine, blood urea nitrogen, and cystatin C, are unable to reflect the split renal injury in the presence of a normal contralateral kidney. When compared to radionuclide dynamic renal imaging, the commonly used method to detect split renal function, CEUS, does not expose the patient to radiation. The contrast agent used for CEUS, sulfur hexafluoride, has a high safety profile for use in humans [18]. It is excreted through the lungs [19] and no renal toxicity is found after the injection of sulfur hexafluoride. CEUS is safe for patients even with renal insufficiency and can be used whenever needed.

We also evaluated the accuracy of the CEUS results by comparing them with the acute tubular necrosis rate found in the specimen 24 h after IRI and we found that both were significantly associated with each other. Acute tubular necrosis can be observed in hematoxylin and eosin and periodic acid-Schiff staining after renal IRI and it is widely used to reflect the severity of kidney injury. Cellular changes can range from minimal alterations to severe cell swelling to individual cell necrosis with denudation of the basement membrane after renal IRI [6]. There may be shedding of both viable and necrotic epithelial cells into the tubular lumen. It can be used to detect whether renal tubules are necrotic or not [6]. We found that the severity of renal IRI increased when the warm ischemia time was more than 20 min. However, even in the same warm ischemia time group, the acute tubular necrosis rate was not exactly the same among mice. It may be that the warm ischemia time is not the only determinant of kidney injury and each individual may have different tolerance to ischemic insult and renal functional recovery ability. CEUS was also used to evaluate anti-angiogenic effects and even treat cancer by synergistic effects in animal models [20–23]. A preliminary study reported that CEUS was used to assess the protective role of a gonadotropin-releasing hormone analog from chemotherapy-induced ovarian damage in a murine model [21]. CEUS with perflubutane in the assessment of anti-angiogenic effects by bevacizumab in a mouse xenografted model was also reported [23]. These preclinical experiments with translational aims have laid an important foundation for the wide application of CEUS in clinical practice.

Although this study well-simulated renal IRI of partial nephrectomy and suggested that the PE and WiPI values can detect renal IRI in an animal model, this study still has some limitations. First, because of the absence of a generally recognized gold standard for kidney injury, we used the renal tubular necrosis rate as the criterion in response to renal IRI. Second, as CEUS is greatly affected by the ultrasonic interface, the selection range, and other factors, we chose the average value of 3 ROIs to reduce the error. Third, the tolerance of the mouse kidney may be different from that of the human kidney. When the basic experimental results are fully applied to clinical settings, they may also be influenced by a variety of clinical factors. Clinical research is needed.

In summary, we showed that renal tubular necrosis occurs after IRI when the ischemia time reaches 20 min in mice. TIC-derived parameters including PE and WiPI values can accurately reflect kidney injury after IRI in mice and may have the potential to predict renal function after partial nephrectomy in humans.

### Supplementary Information

Additional file 1: **Supplementary Figure S1.** The CEUS images and the TIC images after IRI. (a) The CEUS images and the TIC images 1 h after IRI and 24 h after IRI. (b) WiAUC values 1 h (c) and 24 h after IRI. (d) RT values 1 h (e) and 24 h after IRI. (f) TTP values 1 h (g) and 24 h after IRI. (h) WiR values 1 h (i) and 24 h after IRI. The value for each mouse was calculated as the mean of the values of ROI 1, ROI 2 and ROI 3. Ns, no significance, *p* > 0.05; *, *p* < 0.05; **, *p* < 0.01; ***, *p* < 0.001; *ROI* Region of Interest, *RT* Rise time, *TTP* Time to peak, *WiAUC* Wash-in area under the curve, *WiR* Wash-in rate. **Supplementary Figure S2.** The scatter plots of TIC-derived parameters and the renal tubular necrosis rate by HE staining (%) at 24 h after IRI. (a) Scatter plot of WiAUC (a.u) at 1 h after IRI and the renal tubular necrosis rate (%). (b) Scatter plot of WiAUC (a.u) at 24 h after IRI and the renal tubular necrosis rate (%). (c) Scatter plot of RT (s) at 1 h after IRI and the renal tubular necrosis rate (%). (d) Scatter plot of RT (s) at 24 h after IRI and the renal tubular necrosis rate (%). (e) Scatter plot of TTP (s) at 1 h after IRI and the renal tubular necrosis rate (%). (f) Scatter plot of TTP (s) at 24 h after IRI and the renal tubular necrosis rate (%). (g) Scatter plot of WiR (a.u) at 1 h after IRI and the renal tubular necrosis rate (%). (h) Scatter plot of WiR (a.u) at 24 h after IRI and the renal tubular necrosis rate (%).*RT* Rise time, *TTP* Time to peak, *WiAUC* Wash-in area under the curve, *WiR* Wash-in rate.

## Data Availability

The datasets generated and/or analyzed during the current study are not publicly available because the raw data was analyzed by our laboratory instruments and is not publicly available but is available from the corresponding author on a reasonable request.

## Abbreviations

AKI: Acute kidney injury
CEUS: Contrast-enhanced ultrasonography
IRI: Ischemia–reperfusion injury
PE: Peak enhancement
ROI: Region of interest
RT: Rise time
TIC: Time-intensity curve
TTP: Time to peak
WiAUC: Wash-in area under the curve
WiPI: Wash-in perfusion index
WiR: Wash-in rate
