# Supplementary material for: Evaluation of renal ischemia–reperfusion injury using CEUS in mice
Source: Eur Radiol Exp. 2023 Dec 19;7:81. doi: 10.1186/s41747-023-00392-3 (PMC10728419; doi:10.1186/s41747-023-00392-3)
Supplement: Supplementary file 1 — Additional file 1: Supplementary Figure S1. The CEUS images and the TIC images after IRI. (a) The CEUS images and the TIC images 1 h after IRI and 24 h after IRI. (b) WiAUC values 1 h (c) and 24 h after IRI. (d) RT values 1 h (e) and 24 h after IRI. (f) TTP values 1 h (g) and 24 h after IRI. (h) WiR values 1 h (i) and 24 h after IRI. The value for each mouse was calculated as the mean of the values of ROI 1, ROI 2 and ROI 3. Ns, no significance, p > 0.05; *, p < 0.05; **, p < 0.01; ***, p < 0.001; ROI Region of Interest, RT Rise time, TTP Time to peak, WiAUC Wash-in area under the curve, WiR Wash-in rate. Supplementary Figure S2. The scatter plots of TIC-derived parameters and the renal tubular necrosis rate by HE staining (%) at 24 h after IRI. (a) Scatter plot of WiAUC (a.u) at 1 h after IRI and the renal tubular necrosis rate (%). (b) Scatter plot of WiAUC (a.u) at 24 h after IRI and the renal tubular necrosis rate (%). (c) Scatter plot of RT (s) at 1 h after IRI and the renal tubular necrosis rate (%). (d) Scatter plot of RT (s) at 24 h after IRI and the renal tubular necrosis rate (%). (e) Scatter plot of TTP (s) at 1 h after IRI and the renal tubular necrosis rate (%). (f) Scatter plot of TTP (s) at 24 h after IRI and the renal tubular necrosis rate (%). (g) Scatter plot of WiR (a.u) at 1 h after IRI and the renal tubular necrosis rate (%). (h) Scatter plot of WiR (a.u) at 24 h after IRI and the renal tubular necrosis rate (%).RT Rise time, TTP Time to peak, WiAUC Wash-in area under the curve, WiR Wash-in rate. [file 41747_2023_392_MOESM1_ESM.docx]

**Evaluation of renal ischaemia-reperfusion injury using CEUS in mice**

**ELECTRONIC SUPPLEMENTARY MATERIAL**


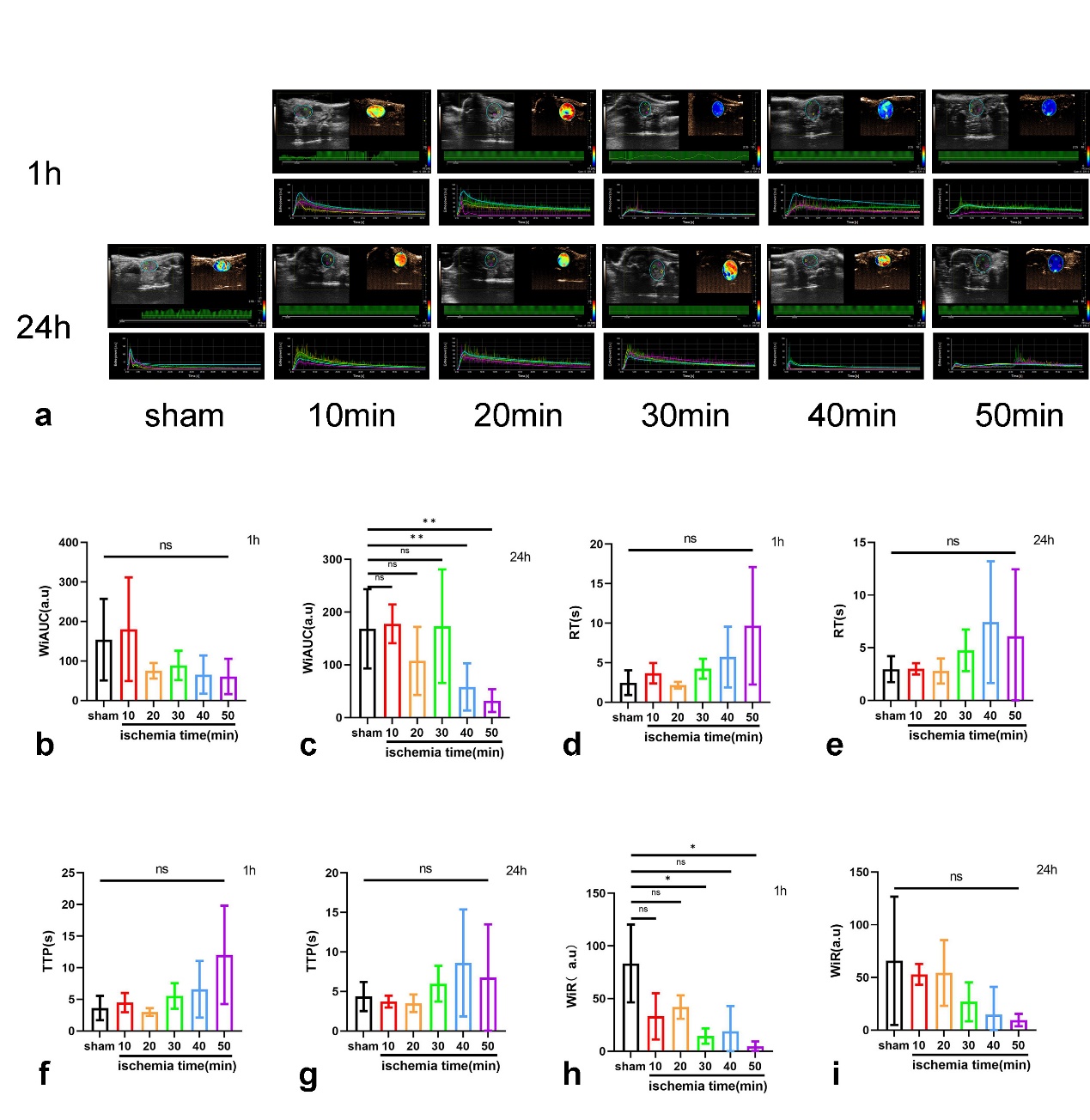


**Supplementary Fig. 1.** The CEUS images and the TIC images after IRI. (a) The CEUS images and the TIC images 1 hour after IRI and 24 hours after IRI. (b) WiAUC values 1 hour (c) and 24 hours after IRI. (d) RT values 1 hour (e) and 24 hours after IRI. (f) TTP values 1 hour (g) and 24 hours after IRI. (h) WiR values 1 hour (i) and 24 hours after IRI. The value for each mouse was calculated as the mean of the values of ROI 1, ROI 2 and ROI 3. Ns, no significance, *p* > 0.05; *, *p* < 0.05; **, *p* < 0.01; ***, *p* < 0.001; *ROI* Region of Interest, *RT* Rise time, *TTP* Time to peak, *WiAUC* Wash-in area under the curve, *WiR* Wash-in rate.


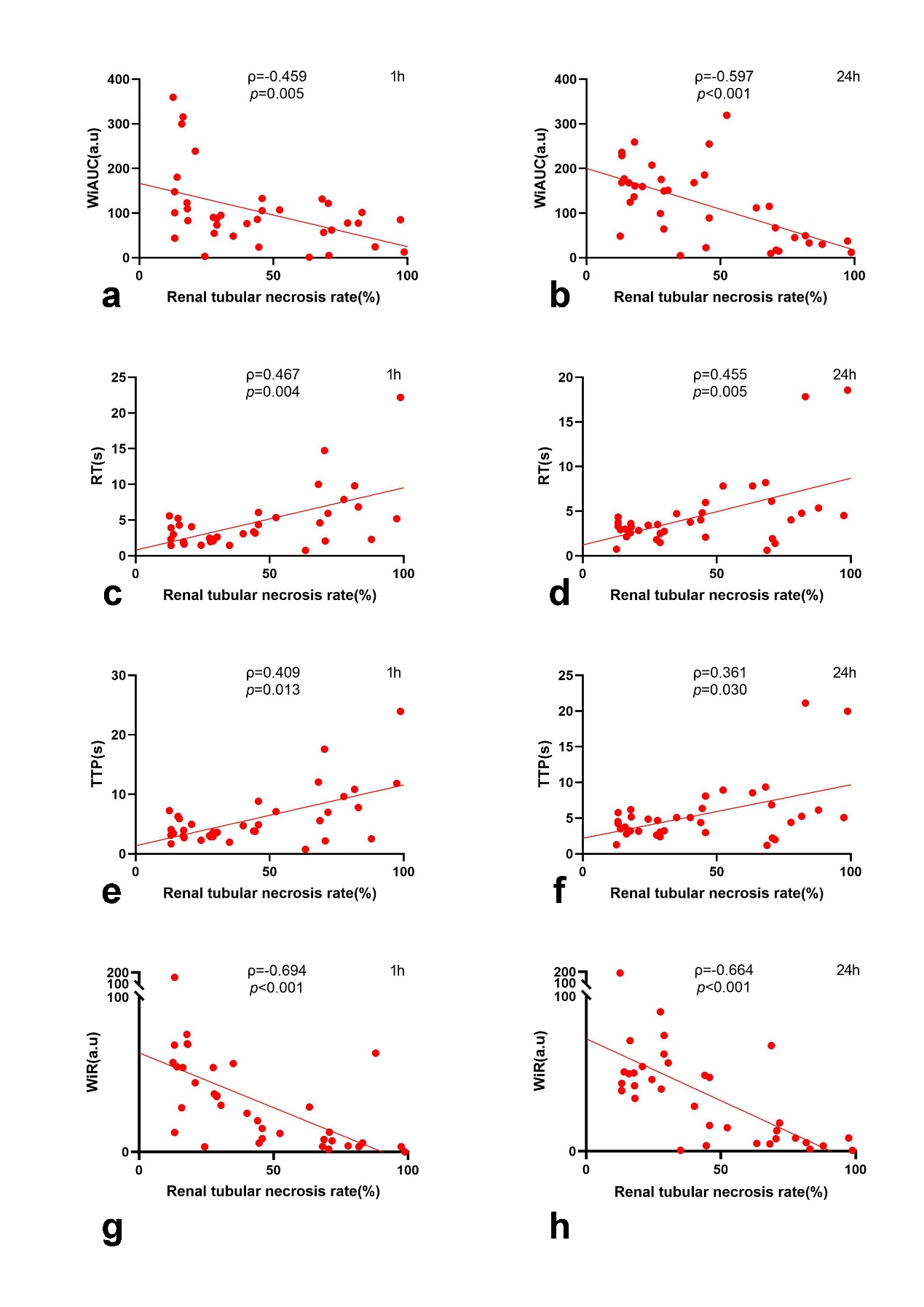


**Supplementary Fig. 2.** The scatter plots of TIC-derived parameters and the renal tubular necrosis rate by HE staining (%) at 24 hours after IRI. (a) Scatter plot of WiAUC (a.u) at 1hour after IRI and the renal tubular necrosis rate (%). (b) Scatter plot of WiAUC (a.u) at 24 hours after IRI and the renal tubular necrosis rate (%). (c) Scatter plot of RT (s) at 1 hour after IRI and the renal tubular necrosis rate (%). (d) Scatter plot of RT (s) at 24 hours after IRI and the renal tubular necrosis rate (%). (e) Scatter plot of TTP (s) at 1 hour after IRI and the renal tubular necrosis rate (%). (f) Scatter plot of TTP (s) at 24 hours after IRI and the renal tubular necrosis rate (%). (g) Scatter plot of WiR (a.u) at 1 hour after IRI and the renal tubular necrosis rate (%). (h) Scatter plot of WiR (a.u) at 24 hours after IRI and the renal tubular necrosis rate (%).*RT* Rise time, *TTP* Time to peak, *WiAUC* Wash-in area under the curve, *WiR* Wash-in rate.
